# Artificial Intelligence-Driven Oncology Clinical Decision Support System for Multidisciplinary Teams

**DOI:** 10.3390/s20174693

**Published:** 2020-08-20

**Authors:** Kyounga Lee, Seon Heui Lee

**Affiliations:** 1 Medical Research Collaborating Center, Seoul National University Hospital, Seoul 03080, Korea; tj720221@snu.ac.kr; 2 Department of Nursing Science, College of Nursing, Gachon University, Incheon 21936, Korea

**Keywords:** clinical decision support system, Watson for Oncology, artificial intelligence, patient satisfaction, patient perception, patient experience

## Abstract

Watson for Oncology (WfO) is a clinical decision support system driven by artificial intelligence. In Korea, WfO is used by multidisciplinary teams (MDTs) caring for cancer patients. This study aimed to investigate the effect of WfO use on hospital satisfaction and perception among patients cared for by MDTs. This was a descriptive study that used a written survey to gather information from cancer patients at a hospital in Korea. The rate of positive change in patient perception after treatment was 86.8% in the MDT-WfO group and 71.2% in the MDT group. In terms of easily understandable explanations, the MDT-WfO (9.53 points) group reported higher satisfaction than the MDT group (9.24 points). Younger patients in the MDT-WfO group showed high levels of satisfaction and reliability of treatment. When WfO was used, the probability of positive change in patient perception of the hospital was 2.53 times higher than when WfO was not used. With a one-point increase in overall satisfaction, the probability of positive change in patient perception of the hospital increased 1.97 times. Therefore, if WfO is used appropriately in the medical field, it may enhance patient satisfaction and change patient perception positively.

## 1. Introduction

Treatment options for cancer patients are becoming more diverse, and treatment guidelines are changing more rapidly; similarly, medical staff need to possess more comprehensive medical information. Medical staff thus spend much time updating their knowledge alongside direct patient care [1]. Oncologists work 56.7 h per week, of which 4.6 h on average are spent updating and maintaining their knowledge of their field [2]. Additionally, as the treatment methods for tumor patients become more diverse and complex, objective evidence and opinions of medical professionals in various fields are necessary to determine the most appropriate treatment methods. Multidisciplinary teams (MDTs), wherein medical professionals from various fields gather to determine the treatment methods for individual tumor patients, have been used in earnest since the 1980s to improve the quality of patient treatment [3]. Although MDTs have the advantage of positively influencing treatment methods for patients, enhancing patient satisfaction, and decreasing the burden of the medical staff, it has been reported that they are inadequate in applying updated medical findings [4,5,6]. Clinical decision support systems (CDSSs) have been developed to solve this problem. CDSSs are artificial intelligence (AI) computer systems used by medical staff to improve healthcare quality by increasing the pace and accuracy [7]. It can identify crucial information from the medical records of patients, find relevant evidence, and suggest appropriate diagnoses and treatment methods. A CDSS has at least two core components: the knowledge base and inference engine. It allows medical staff to retrieve relevant evidence from external resources [8]; thus, a CDSS, which uses a classification model, applies data mining techniques or a knowledge base to assist medical staff in diagnosing the symptoms of patients and finding treatment methods [7]. A CDSS is an AI sensing and smart application needed to reduce the time medical staff spend evaluating evidence-based practice [8].

Watson for Oncology (WfO)—a representative AI-CDSS—is not directly related to patient treatment but provides medical staff with tools to organize and track patient health and medical information and helps facilitate access to medical information. WfO began to be used in earnest at the Memorial Sloan Kettering Cancer Center in 2012. WfO currently offers services for 13 types of cancers: hepatocellular carcinoma, thyroid, ovarian, colon, bladder, esophageal, gastric, breast, cervix, endometrial, prostate, rectal, and lung. WfO provides medical staff with potential evidence-based treatment options by evaluating specific details of patient medical information against a vast amount of clinical evidence (e.g., medical journals, cancer treatment guidelines, drug information, and textbooks), WfO reduces the burden of oncologists by providing them with erudite, updated, and the most recent evidence-based insights. When a medical professional inputs a patient’s clinical information, WfO categorizes treatment options for the patient into three groups, namely “Recommended,” “For Consideration,” or “Not Recommended.” Although WfO suggests treatment methods, it is not directly involved in the patient’s treatment; the final choice is made by the medical staff in consultation with the patient within the scope of the evidence based on national approved treatments. In the future, the group that developed WfO aims to cover approximately 80% of cancer incidence worldwide; however, currently, WfO is used in about 15 countries, including Thailand, India, China, and Korea. It was introduced in Thailand in 2014, India in 2015, and China in 2016. It was first used in Korea in September 2016 at G Hospital; afterward, its use expanded to eight other hospitals [9]. G Hospital, which introduced WfO in Korea, uses WfO in MDTs for cancer patients. In Korea, WfO is classified as software—either providing medical staff with a framework for organizing and tracking the health and medical information of patients or allowing for easy access to medical information—and not as a medical device.

Studies on WfO frequently investigate the concordance rate between treatment method selection by WfO and by tumor boards. A Thai study of breast, rectal, stomach, and lung cancer patients reported a concordance rate of 83% [10] and Indian and Chinese studies on breast cancer patients found concordance rates of 93% and 69.4%, respectively [1,11]. Research in Korea has found concordance rates ranging from 87.7–96.0% [12,13].

While studies on the accuracy of WfO have been conducted, studies examining patients’ experience and satisfaction regarding WfO are rare. Hence, as the use of AI technology is increasing in health care, there is a need to examine patients’ experiences with and perceptions of this technology. Given the increasing interest in the quality of medical services and more emphasis being put on patient-centered medical services globally, it is imperative to evaluate patients’ experiences of hospitals [14,15]. Patient experience can be measured by patient satisfaction, which is a crucial factor in the outcome of patient treatment and an indicator of the quality of health care; as such, the efficacy of WfO can be evaluated through patient perception and satisfaction. Previously, overall patient satisfaction with medical care was measured using a single question. However, detailed measurement tools based on specific experiences of the patient have recently been created. The Medical Expenditure Panel Survey (MEPS) and Hospital Consumer Assessment of Healthcare Providers and Systems (HCAHPS) evaluate patient satisfaction based on patient experience (e.g., careful attention from medical staff, easily understandable explanations, sufficient care time, and respect for the patient). Data from the MEPS suggest that patient experience has a significant relationship with patients’ health resource utilization and healthcare expenditure [16]. Meanwhile, data from HCAHPS surveys revealed that the patient’s satisfaction with the hospital is positively related to the patient’s perception of hospital care [14]. Currently, there is limited empirical knowledge of the patient’s perspective on healthcare aspects that contribute to good patient experience and how patient feedback informs healthcare service development [17].

Therefore, in this study, we assessed patient satisfaction with and perception of a hospital, based on specific experiences and investigated how WfO affects these variables. We intended to analyze patients’ experiences with an AI oncology CDSS and suggest future courses of action.

## 2. Materials and Methods

### 2.1. Study Design and Data Collection

This was a preliminary descriptive study investigating the effects of WfO use on MDT-treated patients’ satisfaction with and perception of the treating hospital. The participants were cancer patients who received treatment from MDTs at G Hospital from March to September 2018. G Hospital is private university hospital. This study was approved by the Clinical Research Ethics Committee of G University (GDIRB2018-109). We explained the purposes and methods of this study to the patients and conducted a written survey among 293 participants who voluntarily agreed to the survey. The final analysis included data from 285 people; eight individuals were excluded due to missing data.

### 2.2. Patient Satisfaction and Perception

We investigated patient satisfaction and perception using structured questionnaires. Questions were created based on literature reviews and information collected from a preliminary meeting with the MDTs. Questions included in the survey covered the following points: general characteristics of the patient, the use of WfO, the difference between the general treatment and WfO, change in the patient’s perception of the hospital after treatment, patient’s satisfaction, and the reliability of treatment methods. General characteristics of patients included sex, age, whether he/she was a new patient, disease stage, and type of cancer. Participants were classified as patients with MDTs using WfO (MDT-WfO) or patients with MDTs that did not use WfO (MDT). MDT-WfO or MDT groups were determined according to the doctor’s preference, since hospitals guarantee the doctor’s choice of medical treatment. Differences between groups were investigated through a yes-or-no question asking whether the treatment provided by an MDT differed from traditional treatment, which is a single doctor dedicated to patient care. We analyzed the satisfaction score relative to the clinical characteristics of the patient, since these factors may have been correlated with one another. Changes in patients’ perception of the hospital after treatment were classified as a positive change, negative change, or no change. Patient satisfaction consisted of overall satisfaction with treatment and satisfaction with specific domains—including medical facilities, clarity of explanations from the medical staff, attentive listening by the medical staff, and sufficient treatment time. The reliability of treatment methods was measured by the level of the patient’s confidence in the final treatment methods. Satisfaction and reliability were measured on a scale of 1 to 10 points, with higher points indicating higher satisfaction and reliability.

### 2.3. Data Analysis

We analyzed patients’ satisfaction with and change in perception of the hospital relative to patient characteristics and whether or not WfO was used. General characteristics were presented as frequencies and percentages. The χ^2^ and Fisher’s exact tests measured the change in the patients’ perception of the hospital, as well as the difference between general medical treatment and treatment from an MDT relative to two factors: general patient characteristics and whether WfO was used or not. Patient satisfaction and the reliability of treatment methods were analyzed using a *t*-test, ANOVA, and Kruskal–Wallis test. A logistic regression analysis was performed to identify the factors affecting change in patient perception to bring about either a positive change (1) or no change (0). In the regression analysis, only variables with *p* < 0.05 or less were examined using univariate analysis. The Hosmer–Lemeshow test was used to test the goodness of fit for logistic regression models. SPSS for Windows version 23.0 (IBM Corp., Armonk, NY, USA) was used for all statistical processing.

## 3. Results

### 3.1. General Characteristics of Patients

Out of the 285 patients who participated in this study, 129 (45.3%) received treatment from an MDT using Watson for Oncology (MDT-WfO) and 156 (54.7%) received treatment from a general MDT. The sample included 253 (88.8%) new patients who had never received treatment from an MDT before. There were 125 men (43.9%) and 160 women (56.1%). The average age of the participants was 60.6 ± 12.9 years. Among the participants, 69 (24.2%) participants were in disease stage 1, 54 (18.9%) in stage 2, 84 (29.5%) in stage 3, and 78 (27.4%) were in stage 4. The most common cancer type was colorectal cancer (*n* = 108; 37.9%), followed by gynecological cancer (*n* = 55; 19.3%), breast cancer (*n* = 48; 16.8%), lung cancer (*n* = 36; 12.6%), liver cancer (*n* = 14; 4.9%), gastric cancer (*n* = 13; 4.6%), and thyroid cancer (*n* = 11; 3.9%). There were no differences in sex and age ratios between MDT-WfO patients and MDT patients. However, there were differences in terms of patient visit (first visit or revisit), disease stage, and cancer type. The MDT-WfO group had many first visit patients. The most common disease stage was stage 3, while the least common was stage 4. Conversely, among those in the MDT groups, stage 4 was the most common, while stage 2 was the least common. In the MDT-WfO group, colorectal cancer was the most common, followed by breast cancer. Colorectal cancer was also the most common in the general MDT group, followed by gynecological cancer; breast cancer was the least common (Table 1).

### 3.2. Patient Perception

Two hundred and twenty participants (77.2%) reported that the treatments they received (MDT-WfO or MDT) were different from treatment from a general single doctor. There was no significant difference in the responses according to the type of MDT or characteristics of the participants. Change in patient perception of the hospital after treatment was categorized as a positive change, negative change, or no change. None of the participants reported negative changes, while 223 (78.2%) reported positive changes and 62 (21.8%) reported no change. The change in perception according to the type of MDT was statistically significant (χ^2^ = 10.183; *p* = 0.001). Out of 129 MDT-WfO participants, 112 (86.8%) answered that their perception of the hospital changed positively post treatment; meanwhile, 111 out of 156 (71.2%) participants in the MDT group reported positive changes in perception. Relative to sex, significantly more women than men reported that they had a positive change in perception of the hospital after treatment (χ^2^ = 5.102; *p* = 0.024). As for cancer type, the rate of positive change in perception of the hospital was the highest among breast cancer patients and was found to be statistically significant (χ^2^ = 15.254; *p* = 0.018) (Table 2).

### 3.3. Patient Satisfaction and Reliability 

Out of 10 points, the average overall satisfaction with treatment was 9.42 ± 1.02 points. Satisfaction was the highest in terms of attentive listening by medical staff (9.49 ± 1.02 points) and sufficient treatment time (9.49 ± 1.01 points). The average reliability of the treatment methods was 9.44 ± 1.02 out of 10. When analyzed according to the treatment group, it was found that the MDT-WfO group had higher overall satisfaction than the MDT group. In particular, satisfaction with easily understandable explanations was significantly higher (*t* = 2.136; *p* = 0.034) in the MDT-WfO group (9.53 ± 0.98 points) than in the MDT group (Table 3).

### 3.4. Patient Satisfaction and Reliability within the Treatment Group

**Figure 1:**
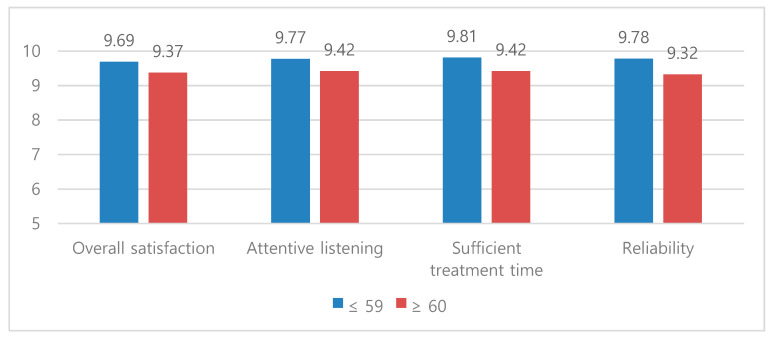
Patient satisfaction and reliability by age in the MDT-WfO group.

Compared to those 60 years old and older, patients 59 years old and younger in the MDT-WfO group were found to have significantly higher satisfaction with medical staff listening and sufficient treatment time and higher overall satisfaction, as well as a significantly higher reliability of treatment methods (Table 4). In the MDT group, the reported satisfaction and reliability of patients 60 years old and older were higher than the group 59 years old and younger; this difference was not significant (Figure 1).

### 3.5. Effect of Patient Satisfaction and WfO on Change in Patient Perception

The univariate analysis determined that WfO use, sex, patient satisfaction, and the reliability of treatment significantly affected patient perception. In the multivariate model, in which all eight variables were used as independent variables, overall satisfaction and the use of WfO led to positive changes in patient perception of the hospital (Table 5). When using WfO, the probability of positive change in perception of the hospital was 2.53 times higher (95% *CI* 1.29–4.97; *p* = 0.007). Finally, when the overall satisfaction increased by a point, the probability of positive change in patient perception of the hospital increased 1.97 times (95% *CI* 1.17–3.32; *p* = 0.011).

## 4. Discussion

Evaluating the satisfaction and experiences of patients is vital in understanding and enhancing the quality of medical care [18]. Technology has improved the efficiency and quality of healthcare services; this is exemplified by the use of systems such as CDSSs and other AI devices to enhance medical staff performance and patient experience [7]. A patient’s satisfaction and experience with their treatment is not only a crucial indicator of the quality of care received by the patient but also influences a patient’s adherence to treatment and thereby affects treatment outcome [19]. Favorable patient satisfaction and experiences increase the likelihood of the patient being faithful to treatment, resulting in positive health outcomes; thus, patient satisfaction and experience can be an indicator for the indirect measurement of the quality of medical care. Patient satisfaction and experience are also related to the degree to which the medical staff meet patient expectations. Therefore, medical staff must consider how to determine and execute priorities to efficiently fulfill patient expectations [20]. In this study, we investigated what patients expected from MDTs using WfO by analyzing patient perception and patient satisfaction. We suggest that patient expectations be treated as foundations for future studies focused on patient-centered care. The main findings of this study can be divided into the following three sections.

First, the analysis of the patients’ perceptions of the hospital after treatment showed that the change in perception was significantly more positive in patients in the MDT-WfO group than in patients in the general MDT group. Therefore, it can be said that the use of WfO has a positive effect on patient satisfaction and perception of the hospital. Analyses based on sex and cancer type revealed that patients who were women or had breast cancer had higher rates of positive perception change. Additionally, logistic regression analysis identified factors affecting the changes in patient perception of the hospital. Univariate analysis showed a significant relationship between patient perception and WfO use, sex, patient satisfaction, and reliability of treatment methods. However, after controlling variables, the multivariate analysis found that only WfO use and overall patient satisfaction had significant effects on patient perception. The probability of a positive change in patient perception of the hospital increased 2.3 times when WfO was used. Positive perception can continuously prompt patients to return to the hospital, bring about a positive clinical outcome, and enhance patient safety by increasing the continuity of treatment [21]. In this study, the use of WfO was found to be the most influential factor in changing patient perception, thus proving the value of WfO in improving clinical outcomes.

Second, satisfaction with easily understandable explanations showed statistically significant differences between groups. Using WfO can help medical professionals explain treatment options to their patients. Science is rapidly changing, thus increasing the number of treatment options available; however, medical staff often have difficulty obtaining the latest information. Even experts such as oncologists tend to have knowledge limited to the treatment of specific cancers, thus making it difficult for them to suggest a variety of novel treatments [22]. WfO allows medical professionals to explain treatments to their patients based on up-to-date information. Listening to explanations based on the most recent scientific evidence may improve patients’ understanding of their disease and treatments for it. It is of utmost importance for patients to be involved in their care and receive the right amount of information [17], since patient satisfaction increases when sufficient information is received and when psychosocial needs related to treatment are met [19,23]. A study that analyzed data from the MEPS in the United States suggests that poor satisfaction among patients is related to increased use of the emergency department rather than the outpatient clinic [24]. One plausible explanation could be that, when satisfaction with hospitals and medical staff is low, patients often do not come to the hospital and tend to not follow the prescriptions given by their doctor. As such, time may be lost before receiving appropriate treatment. Patients then experience complications of the illness that may necessitate immediate attention only obtainable from an emergency department. Therefore, it is necessary to try to improve patient satisfaction. Given that MDT-WfO leads to higher patient satisfaction and positive perception compared to general MDT, using WfO may facilitate appropriate treatment among patients, which, in turn, may lead to positive health outcomes.

Third, WfO can have a positive impact on younger patients. In a meta-analysis that examined the relationship between patient satisfaction and sociodemographic characteristics, age showed the strongest correlation with satisfaction. Generally, satisfaction among elderly patients has been reported to be high [25]. However, the results of this study found that younger patients from the MDT-WfO group showed high satisfaction and reliability of treatment methods. Thus, WfO can raise compliance with medical treatment in younger patients with low treatment satisfaction. A study wherein participants were divided by age into two groups—younger and older than 55 years old—suggests that the information provided by the medical staff is associated with treatment dissatisfaction in patients under 55 [19]. With the help of WfO, medical staff can provide patients with scientific data and sufficient information and explanations; this may improve overall patient satisfaction and reliability of treatment in younger patients.

In sum, among patients receiving treatment from MDTs, WfO is crucial in improving patient satisfaction and positively changing patient perception of the hospital. Previous WfO-related studies focused mostly on the concordance rates between treatment methods chosen by medical staff and those by WfO and how these concordance rates served as both an indicator of the accuracy of WfO and an essential factor influencing patient outcomes. The accuracy of treatment is medically important and precedes patient satisfaction and perception. Previous studies on the accuracy of WfO indicate that a limitation of WfO is its inability to consider certain characteristics of the individual and their environment and culture; these factors include insurance coverage, medical guidelines, race, and geographical region. Additionally, blind trust in WfO can pose problems for communication and acceptance of treatment between medical staff and patients [13]. As such, the accuracy of WfO is to be determined. However, while accuracy takes precedence, patient satisfaction and patient perception are also crucial factors that directly and indirectly affect patient outcomes by changing patient compliance and attitudes toward care. This study found that the use of WfO has a positive effect on the empirical aspects of patient experience. As such, the proper use of WfO in healthcare can lead to positive results in the future.

One limitation of this preliminary study was that it was a questionnaire survey conducted among patients of one university hospital. To be able to generalize the findings of this study, future studies must measure patient satisfaction in various settings. Additionally, the data collected in the study were voluntarily self-reported; publication or survivor bias and recall bias may have affected the information presented. A second limitation was the limited characteristics studied—namely sex, age, and clinical characteristics—in reference to the reliability results. In future studies, it may be beneficial to consider other factors, such as levels of patient anxiety and differences in delivery between medical teams. Third, this study only investigated patient satisfaction and perception relative to whether WfO was used or not among patients being treated by MDTs; we lacked data on patient satisfaction prior to the study. Finally, patient satisfaction and perception are not defined clearly in the existing literature; hence, there is still a need to examine various facets of patient experience. As such, further studies on the differences before and after the use of WfO, differences in medical staff performance, and various definitions of patient satisfaction in various settings should be analyzed.

## 5. Conclusions

The results of this study confirmed that WfO influences patient satisfaction and leads to positive patient perception among patients being treated by MDTs. Specifically, WfO use was found to be the most influential factor that could induce positive change in patient perception of the hospital. WfO use was found to increase patient satisfaction and reliability of treatment methods in younger patients, who generally tend to be less satisfied with their treatment. Patient satisfaction and perception have direct and indirect relationships with the patient’s medical results. Therefore, patient outcomes can be improved by employing MDTs with WfO.

## Figures and Tables

**Table 1:** General characteristics of participants.

Categories		Total		MDT-WfO		MDT		χ^2^/t (*p*)
		*n*	%	*n*	%	*n*	%	
Total		285	100.0	129	45.3	156	54.7	-
Sex	Male	125	43.9	52	40.3	73	46.8	1.206 (0.272)
	Female	160	56.1	77	59.7	83	53.2	
Patient visit	First visit	253	88.8	125	96.9	128	82.1	15.618 (<0.001)
	Revisit	32	11.2	4	3.1	28	17.9	
Age	Mean ± S.D. (Min, Max)	60.6 ± 12.9 (22, 94)		60.1 ± 12.2 (29, 86)		61.0 ± 13.4 (22, 94)		−0.595 (0.552)
	≤39	17	6.0	7	5.4	10	6.4	1.519 (0.218)
	40–49	31	10.9	15	11.6	16	10.3	
	50–59	82	28.8	42	32.6	40	25.6	
	Subtotal (≤59)	130	45.7	64	49.6	66	42.3	
	60–69	87	30.5	35	27.1	52	33.3	
	70–79	46	16.1	21	16.3	25	16.0	
	≥80	22	7.7	9	7.0	13	8.3	
	Subtotal (≥60)	155	54.3	65	50.4	90	57.6	
Stage	1	69	24.2	32	24.8	37	23.7	24.572 (<0.001)
	2	54	18.9	28	21.7	26	16.7	
	3	84	29.5	51	39.5	33	21.2	
	4	78	27.4	18	14.0	60	38.5	
Cancer type	Breast	48	16.8	45	34.9	3	1.9	79.368 (<0.001)
	Colorectal	108	37.9	49	38.0	59	37.8	
	Gastric	13	4.6	9	7.0	4	2.6	
	Gynecological	55	19.3	12	9.3	43	27.6	
	Liver	14	4.9	0	0.0	14	9.0	
	Lung	36	12.6	13	10.1	23	14.7	
	Thyroid	11	3.9	1	0.8	10	6.4	

**Table 2:** Difference from general treatment and changes in patient perception of the hospital.

Categories		Treatment Differences		Perception Changes	
		Yes (%)	No (%)	Positive (%)	No Change (%)
Total		220(77.2)	65(22.8)	223(78.2)	62(21.8)
MDT type	MDT-WfO	99(76.7)	30(23.3)	112(86.8)	17(13.2)
	MDT	121(77.6)	35(22.4)	111(71.2)	45(28.8)
	χ^2^(*p*)	0.027(0.870)		10.183(0.001)	
Sex	Male	100(80.0)	25(20.0)	90(72.0)	35(27.0)
	Female	120(75.0)	40(25.0)	133(83.1)	27(16.9)
	χ^2^(*p*)	0.997(0.318)		5.102(0.024)	
Patient visit	First visit	199(78.7)	54(21.3)	201(79.4)	52(20.6)
	Revisit	21(65.6)	11(34.4)	22(68.8)	10(31.3)
	χ^2^(*p*)	2.740(0.098)		1.909(0.167)	
Age	≤59	103(79.2)	27(20.8)	104(80.0)	26(20.0)
	≥60	117(75.5)	38(24.5)	119(76.8)	36(23.2)
	χ^2^(*p*)	0.564(0.453)		0.432(0.511)	
Stage	1	54(78.3)	15(21.7)	52(75.4)	17(24.6)
	2	46(85.2)	8(14.8)	44(81.5)	10(18.5)
	3	64(76.2)	20(23.8)	66(78.6)	18(21.4)
	4	56(71.8)	22(28.2)	61(78.2)	17(21.8)
	χ^2^(*p*)	3.343(0.342)		0.674(0.879)	
Cancer type	Breast	37(77.1)	11(22.9)	45(93.8)	3(6.3)
	Colorectal	86(79.6)	22(20.4)	87(80.6)	21(19.4)
	Gastric	9(69.2)	4(30.8)	10(76.9)	3(23.1)
	Gynecological	45(81.8)	10(18.2)	42(76.4)	13(23.6)
	Liver	10(71.4)	4(28.6)	9(64.3)	5(35.7)
	Lung	25(69.4)	11(30.6)	22(61.1)	14(38.9)
	Thyroid	8(72.7)	3(27.3)	8(72.7)	3(27.3)
	χ^2^(*p*)	3.118(0.794)		15.254 (0.018)	

**Table 3:** Patient satisfaction and reliability.

Categories		Overall Satisfaction	Specific Patient Satisfaction				Reliability
			Facilities	Easy Explanations	Attentive Listening	Sufficient Treatment Time	
Total		9.42 ± 1.02	9.23 ± 1.26	9.37 ± 1.15	9.49 ± 1.02	9.49 ± 1.01	9.44 ± 1.02
MDT type	MDT-WfO	9.53 ± 0.86	9.34 ± 1.19	9.53 ± 0.98	9.59 ± 0.92	9.61 ± 0.92	9.55 ± 0.95
	MDT	9.33 ± 1.13	9.14 ± 1.32	9.24 ± 1.26	9.40±1.09	9.39 ± 1.07	9.35 ± 1.08
	t(*p*)	1.661(0.098)	1.333(0.183)	2.136(0.034)	1.536(0.126)	1.851(0.065)	1.627(0.105)
Sex	Male	9.33 ± 1.19	9.27 ± 1.35	9.31 ± 1.30	9.46 ± 1.12	9.46 ± 1.12	9.46 ± 1.11
	Female	9.49 ± 0.85	9.20 ± 1.19	9.41 ± 1.01	9.51 ± 0.93	9.52 ± 0.91	9.43 ± 0.95
	t(*p*)	−1.27(0.207)	0.477(0.634)	−0.733(0.464)	−0.465(0.642)	−0.520(0.603)	0.318(0.751)
Patient visit	First visit	9.43 ± 1.02	9.27 ± 1.24	9.38 ± 1.16	9.48 ± 1.05	9.49 ± 1.03	9.44 ± 1.04
	Revisit	9.34 ± 1.03	8.97 ± 1.44	9.28 ± 1.08	9.53 ± 0.72	9.47 ± 0.88	9.44 ± 0.88
	t(*p*)	0.435(0.664)	1.401(0.162)	0.455(0.649)	−0.257(0.798)	0.134(0.894)	0.027(0.979)
Age	≤59	9.47 ± 0.97	9.19 ± 1.31	9.38 ± 1.15	9.48 ± 1.07	9.51 ± 1.03	9.49 ± 0.99
	≥60	9.37 ± 1.05	9.26 ± 1.22	9.36 ± 1.15	9.50 ± 0.98	9.48 ± 1.00	9.40 ± 1.05
	t(*p*)	0.786(0.433)	−0.480(0.631)	0.114(0.909)	−0.164(0.870)	0.252(0.801)	0.757(0.450)
Stage	1	9.39 ± 0.86	9.04 ± 1.34	9.17 ± 1.35	9.38 ± 1.07	9.39 ± 1.07	9.30 ± 1.08
	2	9.65 ± 0.68	9.52 ± 0.95	9.46 ± 1.16	9.61 ± 1.04	9.69 ± 0.84	9.59 ± 0.79
	3	9.45 ± 0.83	9.25 ± 1.25	9.52 ± 0.75	9.60 ± 0.64	9.56 ± 0.77	9.52 ± 0.87
	4	9.24 ± 1.43	9.18 ± 1.37	9.31 ± 1.28	9.38 ± 1.25	9.37 ± 1.25	9.37 ± 1.25
	F(*p*)	1.751(0.157)	1.498(0.215)	1.373(0.251)	1.122(0.340)	1.388(0.247)	1.105(0.347)
Cancer type	Breast	9.75 ± 0.53	9.46 ± 0.92	9.71 ± 0.68	9.77 ± 0.42	9.79 ± 0.41	9.60 ± 0.64
	Colorectal	9.45 ± 0.92	9.24 ± 1.21	9.38 ± 1.10	9.46 ± 1.10	9.47 ± 1.03	9.52 ± 0.92
	Gastric	9.54 ± 0.78	9.54 ± 0.78	9.46 ± 0.88	9.54 ± 0.78	9.62 ± 0.77	9.69 ± 0.75
	Gynecological	9.40 ± 0.83	9.15 ± 1.21	9.33 ± 0.96	9.44 ± 0.96	9.45 ± 0.94	9.33 ± 1.02
	Liver	8.50 ± 2.50	8.93 ± 2.16	9.14 ± 1.96	9.21 ± 1.89	9.07 ± 2.06	9.14 ± 1.96
	Lung	9.22 ± 0.99	9.08 ± 1.52	9.17 ± 1.38	9.39 ± 0.96	9.36 ± 1.02	9.25 ± 1.23
	Thyroid	9.36 ± 1.03	9.09 ± 1.51	8.82 ± 1.89	9.36 ± 1.21	9.36 ± 1.21	9.27 ± 1.27
	χ2(*p*) *	9.247(0.160)	2.341(0.886)	6.818(0.338)	4.077(0.666)	5.058(0.536)	4.470(0.613)

* Kruskal–Wallis test.

**Table 4:** Patient satisfaction and reliability within treatment groups.

Categories		Overall Satisfaction		Specific Patient Satisfaction								Reliability	
				Facilities		Easy Explanations		Attentive Listening		Sufficient Treatment Time			
		MDT-WfO	MDT	MDT-WfO	MDT	MDT-WfO	MDT	MDT-WfO	MDT	MDT-WfO	MDT	MDT-WfO	MDT
Sex	Male	9.37 ± 0.99	9.30 ± 1.32	9.35 ± 1.31	9.22 ± 1.39	9.40 ± 1.18	9.25 ± 1.39	9.52 ± 1.06	9.41 ± 1.16	9.54 ± 1.07	9.40 ± 1.16	9.50 ± 1.11	9.44 ± 1.12
	Female	9.64 ± 0.74	9.35 ± 0.93	9.34 ± 1.11	9.07 ± 1.26	9.61 ± 0.83	9.23 ± 1.13	9.64 ± 0.81	9.40 ± 1.02	9.66 ± 0.80	9.39 ± 0.99	9.58 ± 0.83	9.28 ± 1.04
	t(*p*)	−1.775 (0.078)	−0.265 (0.791)	0.040 (0.968)	0.694 (0.489)	−1.096 (0.276)	0.087 (0.930)	−0.712 (0.478)	0.076 (0.939)	−0.0748 (0.456)	0.068 (0.946)	−0.493 (0.623)	0.933 (0.352)
Patient visit	First visit	9.52 ± 0.87	9.34 ± 1.14	9.35 ± 1.19	9.19 ± 1.28	9.53 ± 0.99	9.23 ± 1.29	9.59 ± 0.92	9.38 ± 1.16	9.61 ± 0.93	9.38 ± 1.10	9.54 ± 0.96	9.34 ± 1.11
	Revisit	9.75 ± 0.50	9.29 ± 1.08	9.00 ± 1.15	8.93 ± 1.49	9.50 ± 1.00	9.25 ± 1.11	9.50 ± 1.00	9.54 ± 0.69	9.75 ± 0.50	9.43 ± 0.92	9.75 ± 0.50	9.39 ± 0.92
	t(*p*)	−0.879 (0.434)	0.220 (0.827)	0.600 (0.589)	0.854 (0.399)	0.055 (0.959)	−0.065 (0.948)	0.182 (0.867)	−0.967 (0.337)	−0.539 (0.621)	−0.230 (0.819)	−0.779 (0.482)	−0.247 (0.806)
Age	≤59	9.69 ± 0.59	9.26 ± 1.21	9.44 ± 1.07	8.95 ± 1.48	9.66 ± 0.70	9.11 ± 1.42	9.77 ± 0.50	9.20 ± 1.36	9.81 ± 0.43	9.21 ± 1.32	9.78 ± 0.49	9.21 ± 1.26
	≥60	9.37 ± 1.04	9.38 ± 1.07	9.25 ± 1.30	9.28 ± 1.17	9.40 ± 1.20	9.33 ± 1.12	9.42 ± 1.17	9.56 ± 0.81	9.42 ± 1.20	9.52 ± 0.82	9.32 ± 1.21	9.46 ± 0.91
	t(*p*)	2.145 (0.034)	−0.658 (0.512)	0.913 (0.363)	−1.521 (0.130)	1.490 (0.139)	−1.118 (0.265)	2.218 (0.029)	−1.907 (0.059)	2.513 (0.014)	−1.684 (0.095)	2.822 (0.006)	−1.400 (0.164)
Stage	1	9.69 ± 0.64	9.14 ± 0.95	9.41 ± 1.01	8.73 ± 1.52	9.53 ± 1.05	8.86 ± 1.51	9.63 ± 0.83	9.16 ± 1.21	9.66 ± 0.83	9.16 ± 1.21	9.56 ± 0.76	9.08 ± 1.26
	2	9.57 ± 0.84	9.73 ± 0.45	9.39 ± 1.17	9.65 ± 0.63	9.54 ± 1.10	9.38 ± 1.24	9.61 ± 1.07	9.62 ± 1.02	9.68 ± 1.02	9.69 ± 0.62	9.57 ± 0.96	9.62 ± 0.57
	3	9.47 ± 0.86	9.42 ± 0.79	9.31 ± 1.29	9.15 ± 1.20	9.55 ± 0.81	9.48 ± 0.67	9.63 ± 0.66	9.55 ± 0.62	9.61 ± 0.75	9.48 ± 0.80	9.57 ± 0.92	9.45 ± 0.79
	4	9.33 ± 1.19	9.22 ± 1.50	9.22 ± 1.31	9.17 ± 1.40	9.44 ± 1.20	9.27 ± 1.31	9.39 ± 1.38	9.38 ± 1.22	9.44 ± 1.34	9.35 ± 1.23	9.44 ± 1.34	9.35 ± 1.23
	F(*p*)	0.774 (0.511)	1.774 (0.154)	0.116 (0.950)	2.603 (0.054)	0.050 (0.985)	1.665 (0.177)	0.332 (0.802)	1.132 (0.338)	0.268 (0.849)	1.378 (0.252)	0.085 (0.968)	1.411 (0.242)
Cancer type	Breast	9.78 ± 0.47	9.33 ± 1.15	9.51 ± 0.89	8.67 ± 1.15	9.80 ± 0.40	8.33 ± 2.08	9.78 ± 0.42	9.67 ± 0.58	9.80 ± 0.40	9.67 ± 0.58	9.64 ± 0.61	9.00 ± 1.00
	Colorectal	9.35 ± 1.01	9.54 ± 0.84	9.16 ± 1.36	9.31 ± 1.07	9.33 ± 1.23	9.42 ± 0.99	9.47 ± 1.19	9.46 ± 1.02	9.49 ± 1.21	9.46 ± 0.86	9.53 ± 1.08	9.51 ± 0.77
	Gastric	9.67 ± 0.71	9.25 ± 0.96	9.89 ± 0.33	8.75 ± 0.96	9.89 ± 0.33	8.50 ± 1.00	9.89 ± 0.33	8.75 ± 0.96	10.00 ± 0.00	8.75 ± 0.96	10.00 ± 0.00	9.00 ± 1.15
	Gynecological	9.75 ± 0.62	9.30 ± 0.86	9.50 ± 0.80	9.05 ± 1.29	9.58 ± 0.79	9.26 ± 1.00	9.58 ± 0.79	9.40 ± 1.00	9.67 ± 0.65	9.40 ± 1.00	9.58 ± 0.79	9.26 ± 1.07
	Lung	9.08 ± 1.26	9.30 ± 0.82	8.85 ± 1.82	9.22 ± 1.35	9.00 ± 1.47	9.26 ± 1.36	9.15 ± 1.21	9.52 ± 0.79	9.08 ± 1.26	9.52 ± 0.85	8.92 ± 1.55	9.43 ± 0.99
	Thyroid	9.00 ± 0.00	9.40 ± 1.07	10.00 ± 0.00	9.00 ± 1.56	10.00 ± 0.00	8.70 ± 1.95	10.00 ± 0.00	9.30 ± 1.25	10.00 ± 0.00	9.30 ± 1.25	10.00 ± 0.00	9.20 ± 1.32
	χ2(*p*) *	8.494 (0.131)	4.184 (0.652)	4.336 (0.502)	4.497 (0.610)	6.969 (0.223)	6.766 (0.343)	5.342 (0.376)	4.106 (0.662)	8.268 (0.142)	3.870 (0.694)	7.226 (0.204)	3.580 (0.733)

* Kruskal–Wallis test.

**Table 5:** Results of logistic regression analyses for perception change.

		Crude			Adjusted		
		*OR*	95% *CI*	*p*-Value	*OR*	95% *CI*	*p*-Value
MDT Type (MDT-WfO)		2.67	1.44~4.95	0.002	2.53	1.29~4.97	0.007
Sex (female)		1.92	1.08~3.38	0.025	1.76	0.94~3.31	0.077
Overall satisfaction		2.23	1.63~3.05	<0.001	1.97	1.17~3.32	0.011
Specific satisfaction	Facilities	1.49	1.22~1.83	<0.001	0.87	0.56~1.35	0.534
	Easy explanations	1.80	1.41~2.30	<0.001	1.40	0.93~2.09	0.104
	Attentive listening	1.84	1.38~2.44	<0.001	0.92	0.38~2.22	0.857
	Sufficient treatment time	1.90	1.43~2.52	<0.001	1.08	0.39~2.99	0.877
Reliability		1.83	1.39~2.41	<0.001	0.99	0.46~2.11	0.981

*OR* = odds ratio; *CI* = confidence interval.
